# Does overcrowding and health insurance type impact patient outcomes in emergency departments?

**DOI:** 10.1186/2191-1991-3-25

**Published:** 2013-11-12

**Authors:** Pedro de Araujo, Maroula Khraiche, Andrea Tukan

**Affiliations:** 1 Department of Economics and Business, Colorado College, 14 E. Cache La Poudre St., Colorado Springs, CO 80903, USA

**Keywords:** Emergency Department, Overcrowding, Instrumental Variable Probit Model

## Abstract

We examine the impact of Emergency Department (ED) overcrowding on wait times and patient outcomes using a unique cross section of about 32,000 patients for an ED located in the Southwestern United States. We construct a measure of a patient’s outcome and estimate the extent to which it is worsened by long waits in the ED. We find that waiting at an ED due to overcrowding tends to generate a negative outcome for all patients. We also find that this negative outcome is larger for those on Medicaid or who have no insurance and smaller for those with private insurance or Medicare.

**JEL Classification Codes:**

I12; I13

## Background

The 1986 Emergency Medical Treatment and Labor Act requires that Emergency Departments (EDs) evaluate any patient who arrives at their door regardless of their ability to pay. As a result, EDs have become the safety nets of the health care system and an important part of demand for health care services [1]. This is particularly true for those individuals who are uninsured and for those with no other option for care [2]. As the demand for emergency services (along with their cost [3]) continues to rise, patients at EDs are facing long waits and medical professionals are dealing with increased overcrowding. Both conditions could be a threat to the continuing viability of U.S. EDs as health care providers [2].

Researchers have long documented the extent of ED overcrowding in the United States. Derlet et al. [4] surveyed a wide range of EDs (academic, private, urban and rural) across the United States and found that 91% of all respondents reported overcrowding as an issue, with EDs serving larger populations reporting more extreme cases of overcrowding. Similarly, [5,6] found evidence of ED overcrowding in hospitals nationwide.

Narrower studies have also found overcrowding patterns. Grumbach et al. [7] documented long waits and overcrowding at San Francisco General Hospital, and [8] surveyed hospitals in three states (Florida, Texas and New York) and reported similar findings.

Many reasons have been identified as the drivers of overcrowding, and one of the most important is the increase in the complexity of the cases seen, which could be the result of an aging population [4]. Other reasons included staff shortages, laboratory result delays and physical capacity limitation at EDs. Many researchers therefore view these trends in overcrowding at EDs as lingering problems that will continue to exist in the future [2].

The impact of overcrowded conditions on patient health outcomes can be quite detrimental. In severe cases, patients in overcrowded EDs have been known to report delays in diagnosis and in the treatment of time sensitive conditions, often leading to extended pain and suffering [4,8]. In other cases, patients who wait long periods of time and who decide to leave before seeing a physician have ended up needing immediate medical attention. Baker et al. [9] show that 29% of patients who left before seeing a physician needed care within 24 to 48 hours, and 11% were hospitalized within the next week. Similarly, [10] show that patients who left after a long wait without seeing a physician were twice as likely to report that their problem has worsened. Additionally, patients on average report lower satisfaction rates during periods of overcrowding at an ED [11].

Long waits and overcrowding seem to particularly impact low income patients who are at risk of preventable negative outcomes [12], and these are not the only costs that come with overcrowding. Significant revenue losses are borne by hospitals with long waits in their ED [13,14]. Provide a survey of the literature documenting the causes and consequences of ED overcrowding.

This paper contributes to the literature on ED overcrowding by estimating the degree to which long waits can affect a patient’s outcome using a cross section of about 32,000 patients for an ED located in a level-one trauma facility in an urban, low socioeconomic demographic of the Southwestern United States. We define a negative patient outcome as a visit to the ED which ended with death, elopement, leaving against medical advice, or leaving without seeing a doctor. Given that any such visit could be characterized by a shorter time spent at the ED, we instrument for wait times using a measure of ED overcrowding at the time a patient visited. This way, we are able to find the direct effect of long waits on patients’ outcomes in the ED while controlling for their condition and their own demographic traits.

Employing two estimation strategies, a linear probability model and a Probit model, we find that longer waits at an ED due to overcrowding make negative outcomes more likely for all patients. Our conclusions are in line with previous research by [15], who use survey data to show that wait times are an important determinant of the decision to leave without seeing a doctor.

Given the mixed evidence about the most relevant patient characteristics to the decision to leave without treatment [16], our paper further contributes to the literature by evaluating patient factors, aside from wait times, that can lead to a negative outcome at an ED. We find that patients who are male or black are more likely to experience a negative outcome (ie elope, leave against medical advice, or leave without seeing a doctor), while patients who have a primary care doctor experience a reduction in this likelihood. This latter result is in line with previous research by [7], who conclude that patients who seek primary care regularly tend to use the ED for more clinically appropriate reasons and are therefore less willing to leave without seeing a doctor.

Our findings also suggest that insurance status plays a role in determining the likelihood of a negative outcome. More specifically, we find that the likelihood of a negative outcome is higher for those with Medicaid and for those who have no insurance, while the likelihood of a negative outcome is smaller for those with private insurance and for those on Medicare.^a^ These results are in contrast with the conclusions of [15] who find that patient characteristics do not matter for the decision to leave an ED before seeing a doctor, but support those reported by [16].

## Data and descriptive statistics

We use data collected from an ED located in a level one trauma facility in an urban, low socioeconomic demographic of the Southwestern United States. The data obtained was the ED’s scorecard records, which are daily logs of patient flow from January 1, 2011-October 1, 2011. The logs have information on each patient’s check in time, the time they were assessed by a physician, the acuity of the initial complaint^b^, checkout time, and final disposition. The final disposition describes the outcome of the patient’s visit. Patients are typically discharged, admitted to the hospital, transferred to another facility, pronounced dead or they leave without treatment or against medical advice. Additional data was obtained about each patient’s characteristics including race, age, gender, patient’s insurance, and the patient’s primary care physician. Basic summary statistics are provided in Table 1.

**Table 1:** Summary statistics

**Variable**	**Mean**	**Std. Dev**
Age	43	20.36
Male	42.35%	0.49
Medicaid	41.74%	0.49
Private	18.01%	0.38
SelfPay	15.98%	0.37
White	40.33%	0.49
Black	15.62%	0.36
Hispanic	39.12%	0.49
Primary Dr.	66.38%	0.47
**Total Observations**	57959	

In our sample, an average of 219 patients check into the ED everyday. Patients spent on average 3.9 hours in the ED. Before seeing a doctor, patients waited an average of over thirty minutes. The maximum number of hours waited before seeing a doctor was over six hours. On average, 7.8 patients died, eloped, left against medical advice *or* left without seeing a doctor daily. About 16% of the patients in our sample had no insurance and 18% had private insurance. Of those who had no insurance, about 35% were White, 14% were Black and 47.5% were Hispanic. Waits times varied for each demographic group. Examining Table 2, it is evident that those who paid using Medicaid waited the longest and those who had Medicare waited the least. It is also evident that, for any insurance type, Whites waited the least and Hispanics waited the longest. In terms of the outcome of the visit, of those who died, eloped, left against medical advice or left without seeing a doctor, only 34% were white, while the other two thirds were Black, Hispanic or of other ethnicity. These statistics can be found in Table 3.

**Table 2:** Wait time (in hours) by race and insurance

	**All**	**White**	**Black**	**Hispanic**
All	0.5626	0.5077	0.5753	0.6227
Medicare	0.4509	0.4138	0.5063	0.5125
Private	0.5117	0.4746	0.5020	0.5721
Selfpay	0.5996	0.5624	0.6010	0.6295
Medicaid	0.6252	0.5764	0.6360	0.6692

**Table 3:** Demographic patient characteristics

	**% In sample**	**% of Type**		
	**All Groups**	**White**	**Black**	**Hispanic**
Self Pay	11.26%	35.77%	13.66%	47.50%
Medicare	11.58%	52.04%	14.96%	25.95%
Medicaid	42.35%	30.73%	17.88%	44.92%
Private	33.71%	48.02%	14.98%	33.86%
Primary Dr.	24.03%	40.50%	15.72%	38.68%
Left, Eloped or Died	3.45%	34.70%	20.48%	31.66%

Additionally, we find that wait times and the outcome of the visit were different depending on the day of the week. Patients who visited the ED during a weekday waited longer than those who visited the ED during the weekend. However, a higher percentage of patients died, eloped, left against medical advice *or* left without seeing a doctor on weekday than on the weekend. This could be due to the higher cost of waiting associated with a weekday when people generally work compared to a weekend. The statistics can be found in Table 4.

**Table 4:** Wait time and outcome by days of the week

	**Avg Wait Time**	**Std. Dev**	**Avg % of**	**Std. Dev**
	**in Hours**		**Patient center**	
Monday	0.7609	0.0104	4.50 %	0.0029
Tuesday	0.7284	0.0109	5.13 %	0.0033
Wednesday	0.6218	0.0088	3.22 %	0.0026
Thursday	0.5672	0.0080	3.23 %	0.0026
Friday	0.5127	0.0073	3.38 %	0.0027
Saturday	0.3954	0.0057	2.12 %	0.0022
Sunday	0.3858	0.0050	1.84 %	0.0020

In order to understand the significance of the difference in wait times on the the array of groups we have in our sample, the next section shows the results of regression analysis estimating the impact of wait times on patient outcomes, controlling for the demographic characteristics of the patients.

## Methods

In order to estimate the direct impact of wait times on the patient outcome, we control for patient characteristics and the patient’s medical condition at the time of patient check-in. The variables describing patient characteristics include dummies for race, insurance type and whether a patient has a primary doctor, and a continuous variable for the patient’s age. The measure we have for the patient’s medical condition is a rank for the acuity of their condition, with 1 being a condition that requires immediate attention. Therefore, we include a dummy for those with acuity levels of 1 or 2 to capture severe medical conditions. Finally, we include our variable of interest: the patient’s wait time before he or she sees a doctor.

The outcome variable we construct is a dummy variable for whether a patient died, eloped, left against medical advice or left without seeing a doctor. Therefore, a positive coefficient associated with any of the independent variables in our estimation implies a negative health outcome for the patient. Given that those who leave before being treated or evaluated tend to need medical treatment on a later date or experience a worsening in their condition [9], our measure of patient outcome describes both the patient’s wellbeing on a later date and his or her access to medical attention.

We are interested in finding out how a patient’s outcome is affected by long waits at the ED. One strategy, then, is to use the wait time variable directly as an explanatory variable determining patients’ outcomes. In this case, the estimated coefficient for how long the patient waited pins down the impact of wait time on a patient’s outcome. However, given our definition of patient outcome, it might be the case that those who elope, die or leave before seeing a doctor would register shorter time spent at the ED. To test for this possibility, we conduct the Durbin-Wu-Hausman endogeneity test suggested by [17] to check whether the regressor describing wait times is endogenous. As can be seen in Table 5, the test fails to reject the null hypothesis that wait times are exogenous.

**Table 5:** Durbin-Wu-Hausman Endogeneity test

	
Robust score *χ*^2^(1)	= 1.01355 (p = 0.3141)
Robust regression F(1,31346)	= 1.01209 (p = 0.3144)

To deal with this issue of the endogeneity of wait times, we instrument for wait time before seeing a doctor with the average number of patients in the ED at the time the patient checks in which in turn captures the level of crowding at the ED. Our instrumental variable is valid under the assumption that patients’ decision to leave is not driven by the number of people at the ED, but by the wait time faced (which increases when the ED is crowded). This assumption implies that when patients walk into the ED, whether they choose to stay or leave will depend on how long they have to wait (and other considerations such as acuity of condition), rather than the number of other patients they observe. The aforementioned instrumental variable estimation strategy takes care of any endogeneity concerns and uncovers the estimate of the impact on patient’s outcome from overcrowding itself through longer wait. To test the validity of our instrument, we perform the [18] test for weak instruments and our results which are presented in Table 6 show that the test rejects the null hypothesis that our instrument is weak. We also present the first stage estimation results in Table 7.

**Table 6:** Stock and Yogo (2002) Test for Weak Instruments

**Minimum eigenvalue statistic = 1128.45**				
**Critical Values -*H***_***0***_**:Instruments are weak**	**10%**	**15%**	**20%**	**25%**
2SLS Size of nominal 5% Wald test	16.38	8.96	6.66	5.53
LIML Size of nominal 5% Wald test	16.38	8.96	6.66	5.53

**Table 7:** First-stage regression summary statistics

**Variable**	***R***^***2***^	**Robust F(1,31347)**	**Prob *>*** **F**
Time before Dr.	0.0664	876.022	0.0000

## Results

The estimation results for traditional ordinary least squares and a two-stage least squares (where time waited is instrumented for using overcrowding) are presented in Table 8. Given the binary nature of our outcome variable, we also estimate a standard Probit model and an instrumental variable Probit model. The marginal effects for the Probit model and the IV Probit are also reported in Table 8.^c^ In the two-stage least squares estimation and the IV Probit, the first-stage estimation shows that our instrument is significant (refer to Table 7 for test statistics).

**Table 8:** Regression results - overcrowding as IV

	**Probit**	**IV Probit**	**OLS**	**2SLS**
Time before Dr.	0.011403 ***	0.019043 **	0.015416 ***	0.022015 ***
	0.001101	0.008156	0.001871	0.006755
Private Ins.	-0.013445 ***	-0.014009 ***	-0.013687 ***	-0.013342 ***
	0.001696	0.002031	0.001949	0.001945
Medicare	-0.007951 **	-0.008128 **	-0.010053 ***	-0.009675 ***
	0.002317	0.002546	0.002916	0.002916
No Insurance	0.003865 *	0.004258 *	0.005153 *	0.005292 *
	0.002496	0.002672	0.003225	0.003224
Black	0.008312 **	0.008498 *	0.008167 *	0.007976 *
	0.004660	0.004881	0.004255	0.004250
Hispanic	-0.004074	-0.004749	-0.004616	-0.005049
	0.003497	0.003755	0.003763	0.003780
White	0.001080	0.001027	0.000757	0.000659
	0.003697	0.003907	0.003836	0.003837
Male	0.009814 ***	0.010705 ***	0.010111 ***	0.010428 ***
	0.001649	0.002134	0.001659	0.001732
Primary Dr.	-0.003180 *	-0.003599 *	-0.003446 *	-0.003633 *
	0.001760	0.001889	0.001934	0.001922
Age	-0.000032	-0.000007	0.000004	0.000029
	0.000047	0.000056	0.000042	0.000050
High Acuity	0.072523 ***	0.081609 ***	0.058099 **	0.060079 ***
	0.022305	0.024797	0.018541	0.018440
*Waldχ*^2^	307.05	215.89		178.85
*F*			19.58	

*, ** and *** indicate a 1%, 5% and 10% significance level. Standard errors are robust. The complete sample for reported regressions has 31,359 patients.

Under all specifications, longer wait times lead to an increase in the likelihood of negative outcomes for the patients. This effect is larger under an endogenous variable assumption. Also, having insurance (private or Medicare) alleviates some of the negative impact on patients’ outcomes, while having no insurance or paying with Medicaid exacerbates negative outcomes for patients. The results of our IV Probit suggest that, on average, waiting an extra hour at the ED increases the likelihood of a negative outcome by 1.9%. Compared to being on Medicaid, having private insurance or Medicare *decreases* the likelihood of a negative outcome by.6% and.8%, respectively. Those who have no insurance experience a.14% *increase* in the likelihood of a negative outcome. These results are of importance given that uninsured patients do not typically have access to health services other than the emergency room [16,19] and typically experience preventable health outcomes that can be addressed with timely attention.

Our findings also show that those with a primary doctor faced a better outcome. This relationship can be explained in several ways. First, those with a primary doctor could represent patients who get regular checkups or who are health conscious, and thus tend to be more patient and less likely to leave against medical advice or without seeing a doctor. Additionally, those with access to primary care tend to use the ED more appropriately (compared to those with no access to primary care) and therefore are less likely to leave without seeing a doctor [7].

Additionally, as expected, having an acute health condition or being older is associated with negative patient outcomes, in part because death is a possible negative outcome. Finally, being male or black increases the likelihood of a negative outcome.

### Robustness checks

We run several robustness checks and extend our model to test the sensitivity of our results. First, we add the average number of doctors per patient per day as another instrument (along with ED overcrowding). Secondly, we construct a dummy as another outcome variable for whether a patient eloped, left against medical advice or left without seeing a doctor (this outcome variable is identical to our original variable but excludes the patients who died). Thirdly, we add controls for the days of the week. We find that our results generally hold for all these robustness checks. We report our findings for the margins of the IV Probit in Tables 9 and 10.

**Table 9:** Robustness checks

	**IV Probit**	**IV Probit**
	**Average Dr. &**	**Outcome Excludes**
	**Overcrowing as IV**	**Death**
Time before Dr.	0.023871 **	0.023329 **
	0.008173	0.008810
Private Ins.	-0.015177 ***	-0.015115 ***
	0.002195	0.002223
Medicare	-0.008323 **	-0.008313 **
	0.002714	0.002686
No Insurance	0.004580 *	0.004542 *
	0.002738	0.002734
Black	0.008966 *	0.008954 *
	0.005044	0.005024
Hispanic	-0.005652	-0.005597
	0.003863	0.003854
White	0.001371	0.001380
	0.004047	0.004027
Male	0.010783 ***	0.010714 ***
	0.002178	0.002217
Primary Dr.	-0.003364 *	-0.003335 *
	0.001950	0.001947
Age	-0.000014	-0.000016
	0.000056	0.000057
High Acuity	-0.001582	-0.001541
	0.001784	0.001781
*Waldχ*^2^	214.47	212.98

*, ** and *** indicate a 1%, 5% and 10% significance level. Standard errors are robust. The complete sample for reported regressions has 31,359 patients.

**Table 10:** Robustness checks

	**IV Probit**	**IV Probit**
	**(Days of the week controls)**	**(Weekend controls)**
Time before Dr.	0.018495 *	0.017525 **
	0.009713	0.008907
Private Ins.	-0.013979 ***	-0.013934 ***
	0.002044	0.002009
Medicare	-0.007979 **	-0.008047 ***
	0.002534	0.002511
No Insurance	0.004313 *	0.004189 *
	0.002669	0.002642
Black	0.008335 *	0.008473 *
	0.004844	0.004835
Hispanic	-0.004701	-0.004604
	0.003760	0.003725
White	0.001012	0.001081
	0.003886	0.003866
Male	0.010637 ***	0.010476 ***
	0.002203	0.002124
Primary Dr.	-0.003377 *	-0.003541 *
	0.001887	0.001870
Age	-0.000011	-0.000013
	0.000059	0.000057
High Acuity	0.080573 ***	0.079769 ***
	0.025137	0.024866
Monday	0.002701	
	0.004060	
Tuesday	-0.001105	
	0.003871	
Wednesday	0.001881	
	0.003504	
Thursday	0.006232 *	
	0.003264	
Friday	0.007148 **	
	0.003305	
Saturday	0.002150	
	0.003246	
Weekend		-0.0024655
		0.0024646
*Waldχ*^2^	228	221

*, ** and *** indicate a 1%, 5% and 10% significance level. Standard errors are robust. The complete sample for reported regressions has 31,359 patients.

The only change we observe in the results when conducting our robustness checks is that when we exclude death as a negative outcome, acuity becomes insignificant. This might imply that unless the illness is life threatening, the level of acuity will not impact the patient’s decision to leave the ED. We also find that patients are more likely to leave on a weekday (the coefficients are slightly higher for weekdays than for Saturday and the coefficient is negative for Sunday). However, the coefficients for the day of the week controls are only statistically significant for Thursday and Friday.

Our final extension to the original model is to test whether health insurance status exacerbates the effect of wait times on the decision to leave without seeing a doctor by adding interaction terms between each insurance status and wait times. However, we find that when adding controls for days of the week, the interaction terms are insignificant with the exception of the interaction term between wait times and Medicare. We find that the impact of wait times on negative outcomes is reduced when patients are on medicare in some of the cases.

## Discussion

We examine the impact of ED wait times on patient outcome using a unique cross section of about 32,000 patients. We define a negative patient outcome as a visit to the ED ending with death, elopement, leaving against medical advice or leaving without seeing a doctor. Since these types of ED visits may be shortened by a patient’s decision to leave, we instrument for wait times using a measure of ED overcrowding at the time the patient visited the ED. We find that longer waits at an ED increase the likelihood of negative outcomes for all patients. We also find that this negative effect is larger for those with no insurance or who are on Medicaid, and smaller for those with private insurance or who are on Medicare. Our data set does not reveal the reason why health insurance status plays a role in alleviating or exacerbating a negative outcome for patients. One conjecture is that those who have no insurance or are on medicaid use the ED for reasons that are different than those who have private insurance, since those uninsured groups have no other venue for receiving health care services. Also, uninsured groups who do not qualify for medicaid are more likely to have part time jobs that do not offer sick days, and therefore any time spent at the ED may be more costly. Finally, the aforementioned groups may also have little support at home or access to daycare services, and therefore may be less able to spend time away from home. However, our data does not allow us to test for these possibilities.

In terms of solutions, our findings regarding primary health care are promising. In our sample, patients who listed a primary care doctor were less likely to experience a negative outcome. It’s worth noting that 78% of those who listed a primary care doctor had private insurance or Medicare. Therefore, our recommendations are in line with those suggested by [7]. The authors suggest a primary care in-house clinic at the hospital which could accept patients on a next-day appointment basis for certain conditions, and they also suggest creating a mechanism to redirect patients to primary care facilities by helping them make next day appointments. This can reduce the average number of patients at EDs while guaranteeing that access to health care services is available to patients who seek it.

## Endnotes

^a^ This finding is important given that the percentage of people covered by Medicaid in 2011 increased to 16.5%, and the 15.7% of uninsured households in the population are typically characterized as low income households and more importantly, children in poverty are more likely to be uninsured [20].

^b^ As patients arrive at an ED they are typically evaluated by a triage nurse who assesses their condition and gives priority to those with more severe complaints.

^c^ In all estimations, the standard errors reported are robust to heteroskedasticity. Stata is used for all estimations.

## Competing interests

The authors declare that they have no competing interests.

## Authors’ contributions

AT collected data and conducted preliminary analysis. PA and MK conducted further analysis and robustness checks. MK drafted the final version of the paper in dialogue with PA. All authors read and approved the final manuscript.
